# Microbial Carbon Limitation Mediates Soil Organic Carbon Sequestration in Sugarcane–Watermelon Intercropping System

**DOI:** 10.3390/microorganisms13051049

**Published:** 2025-04-30

**Authors:** Lixue Wu, Yue Fu, Tian Zhang, Tingting Sun

**Affiliations:** Guangxi Key Laboratory for Agro-Environment and Agro-Products Safety, College of Agriculture, Guangxi University, Nanning 530004, China; lixuewu2024@163.com (L.W.); fyue0606@163.com (Y.F.); zt21400s@163.com (T.Z.)

**Keywords:** soil respiration, intercropping, rhizosphere, microbial carbon limitation, carbon fractions

## Abstract

Intercropping is an effective approach for enhancing soil organic carbon (SOC) sequestration. However, the effects of intercropping on SOC dynamics and the underlying factors in rhizosphere and bulk soils are still unclear. In this study, we examined the impacts of sugarcane monoculture and sugarcane–watermelon intercropping on soil properties, soil respiration, SOC fractions, and microbial C limitation with continuous two years in 2023–2024 years in the Nala area of Guangxi Province. Our results revealed that intercropping significantly decreased CO_2_/SOC by 25% and microbial C limitation by 21% in the rhizosphere, with more pronounced reductions observed in bulk soil by 33% and 25%, respectively. This means that the intercropping reduced soil respiration and this effect can be offset by the rhizosphere effects. Additionally, the sugarcane–watermelon intercropping increased the contents of mineral-associated organic carbon (MAOC) by 15~18% and particulate organic carbon (POC) by 34~46%. The random forest analysis indicated that enzyme activities (explaining 20~38% of variation) and soil properties (explaining 22% of variation) were the primary drivers of reduced CO_2_ emissions. The PLS-PM showed that intercropping decreased microbial C limitation by influencing soil pH and soil water content (SWC), and then increased MAOC, which finally led to a decline in CO_2_ emissions. Overall, these findings highlight the decreasing CO_2_ emissions during the use of the intercropping system and the importance of microbial C limitation in the soil C cycle via soil respiration and SOC fractions.

## 1. Introduction

Soil respiration is an important process of carbon feedback in the soil–atmosphere system [1]. The increased soil respiration generates a potential risk of more CO_2_ emission into the atmosphere and thereby global warming [2]. Agricultural soil occupies 37% of the global land area and has the most potential impacts on soil organic carbon (SOC) sequestration and climate change [3]. Intercropping is considered a sustainable crop pattern that can improve land productivity and agricultural ecosystem services, for example, soil carbon and nutrient (nitrogen (N) and phosphorus (P)) dynamic changes [4,5]. The reason is the functional reimbursement of various crops to enhance the land equivalent ratio with high water content, air, sunlight, and nutrient use efficiency in the same land [4]. Recent studies have shown that compared with monoculture, intercropping with high crop diversity is affected by many factors, which leads to the reduction in, increase in, or less impact on soil respiration [6,7,8]. Soil respiration is also influenced by soil moisture and soil temperature; higher soil temperature and lower soil moisture mean lower soil CO_2_ emission [1]. However, soil respiration still remains uncertain in response to long-term intercropping systems, which impedes the understanding of soil–atmosphere C cycle responses to agricultural management practices.

This uncertainty is ascribed to the truth that soil respiration is regulated by multiple factors, including SOC fractions, microbial activity, and soil conditions. SOC fractions have different functions and stability [9,10]. For example, mineral-associated organic C (MAOC) is the stable form of soil C and is hard to change with agricultural management practices [11]. Instead, particulate organic C (POC) is the labile form of C and is mostly derived from root C, which is easily decomposed by microbes [12]. The concentrations and ratio of these two C components thus regulate soil respiration. In addition, soil respiration is mainly controlled by the microbial metabolism process via producing different enzymes in the decomposition of soil organic matter (SOM) and soil nutrients [13,14]. For instance, higher C-related enzymes are believed to have stronger microbial demands on C; consequently, more SOM is decomposed, and CO_2_ concentrations in the atmosphere increase [15]. The enzyme activity is controlled by soil pH and nutrient conditions [16,17], which could further affect soil respiration [18]. However, the relative importance of SOC components, microbial activity, and soil conditions for soil respiration under intercropping systems is still unknown, which hinders the prediction of soil respiration and CO_2_ emission in response to diversified cropping systems in agricultural soils.

The rhizosphere, the interface of soil–roots–microbes, has faster turnover rates in soil nutrient cycling and SOC dynamics than those in the bulk soil because of rhizodeposition [19,20]. Given the enrichment in root exudates and the specific microbial community selected by surrounding roots, the rhizosphere is thus expected to have high proportions of labile C and plant-derived C (e.g., POC), and bulk soil is dominated by recalcitrant C (e.g., MAOC) [21]. Our recent study found that intercropping effects on SOC sequestration and components (POC vs. MAOC) differed in rhizosphere and bulk soils [22]. Thus, the changes in microbial communities, activity, and soil nutrients induced by intercropping could alter soil respiration to different extents. However, there is little direct evidence for the differences in soil respiration between the rhizosphere and the bulk soils [23,24,25]. Therefore, exploring soil respiration in both rhizosphere and bulk soils under intercropping systems might help researchers better understand the soil C cycle within root–soil systems.

Sugarcane is the most important cane sugar crop in the world and is largely planted in southwest China [26]. Sugarcane–watermelon intercropping is widely introduced to increase crop yield and revenue [27,28]. Here, we aim to explore and compare soil respiration and the underlying factors under sugarcane–watermelon intercropping systems in rhizosphere and bulk soils. This study aimed to evaluate a quantitative comparison of rhizosphere versus bulk soil respiration dynamics in sugarcane–watermelon intercropping systems, revealing the predominant driver of CO_2_ and understanding how the rhizosphere effect influences the intercropping effect. We hypothesize the following: (1) sugarcane–watermelon intercropping might increase soil respiration due to higher plant diversity under intercropping systems in both rhizosphere and bulk soils [29]; (2) the intercropping-induced changes in soil respiration may be higher in bulk soil than in rhizosphere soil because of higher intercropping effects on microbial activity in bulk soil [24]; (3) soil respiration might be mostly regulated by microbial activity (e.g., enzyme activity), followed by SOC component and soil conditions. This study presents the quantitative comparison of sugarcane–watermelon intercropping effects on soil C dynamics (SOC content, SOC fraction, and soil respiration) between rhizosphere and bulk soils, and reveals the predominant drivers of soil C sequestration.

## 2. Materials and Methods

### 2.1. Study Area and Site Selection

**Figure 1:**
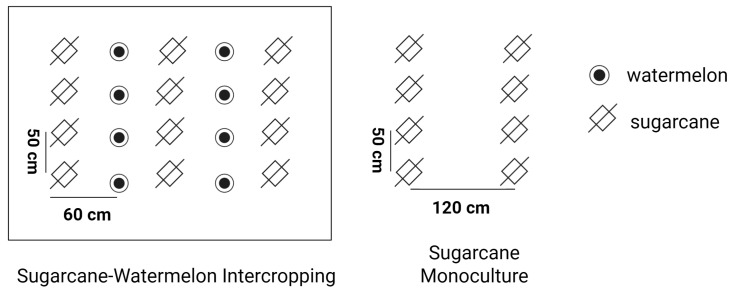
The schematic diagram of sugarcane–watermelon intercropping and monoculture sugarcane.

The long-term field experiment was conducted in the Nala watershed, Kelan Reservoir, Sui County, Guangxi, China (22°20′54″ N, 107°39′44″ E). Nala belongs to the subtropical monsoon humid climate, the annual temperature is between 20.8 and 22.4 °C, and the annual rainfall is about 1200 mm, which is concentrated in March and September. The soil type is red soil, which comes from the parent rock of soil formation, and the parent material mainly includes shale, sandstone, a conglomerate of noncarbonate rock, and Quaternary red soil parent material and ancient sediments. Two treatments were selected in this study, namely sugarcane monoculture and sugarcane–watermelon intercropping. The line spacing for sugarcanes was 1.2 m and that for the sugarcane intercropping watermelon was 60 cm (Figure 1). Sugarcane and watermelon were annually planted in late February, with harvests occurring in December and July, respectively; the overlapping time was March to June. A content of 600 kg ha^−1^ organic fertilizer was used in all treatments each year. After the watermelon harvest, sugarcane used 300 kg ha^−1^ of special compound fertilizer (N/P_2_O_5_/K_2_O = 21:8:16). The yield of sugarcane was 80 t ha^−1^ in both the monoculture and intercropping systems, and the watermelon yield was 13 t ha^−1^ in the intercropping system. When sugarcane was harvested, all experiment plots were incorporated with residual straw to maintain field integrity. The management practices of the experiment were completely in accordance with local agronomic practices.

### 2.2. Soil Sampling

Soils were collected in June in 2023 and 2024. June was a key period for studying the sugarcane–watermelon intercropping effect because the fast growth of sugarcane and high nutrient demands influence SOC dynamics [26]. The samples were taken in each square according to the five-point sampling method. We sampled rhizosphere and bulk soils in sugarcane monoculture and sugarcane–watermelon intercropping. The rhizosphere soil was collected from tightly adhered soil at plant roots after the removal of loosely adhered soil [30]. The bulk soil was collected at a distance of 20 cm from the crops. We collected 24 samples in 2023 (2 soils × 6 replicates in monoculture and intercropping) and 18 samples in 2024 (2 soils × 6 replicates in monoculture and 3 replicates in intercropping). The soil samples were removed from visible stones and root residues and sieved to 2 mm, and the soil was divided into two parts. Subsequently, one part was immediately analyzed for enzyme activity and soil respiration, and the other part was stored at 4 °C for an analysis of soil physiochemical properties.

### 2.3. Soil Physicochemical Properties

The soil water content (SWC) was determined by the weight difference method for calculation. We used a pH meter (Seven Compact S220, Shanghai, China) to measure soil pH (fresh soil/water = 1:5). The dried samples were utilized to determine the soil total nitrogen (TN) content by the Kjeldahl method after digestion by using concentrated H_2_SO_4_. The fresh samples were utilized to extract the soil NO_3_^−^ by 2 mol/L KCl (soil mass/volume = 1:10) [31]. Soil available P (SAP) and total P (TP) were measured by the molybdenum blue method [32]. NO_3_^−^, SAP, and TP were measured using an ultraviolet spectrophotometer (UV2600, Shimadzu, Kyoto, Japan).

### 2.4. Soil Enzyme Activity

For enzyme activity analysis, fresh soil samples (1.5 g) were mixed with Tris buffer (pH = 8). Subsequently, the mixture was subjected to oscillation for 2 min. After the soil slurries were mixed with the substrates, they were incubated at 25 °C in the dark for 3 h. The activities of the β-1,4-glucosidase (BG), acid phosphatase (AP), β-1,4-Nacetylglucosaminidase (NAG), and leucine aminopeptidase (LAP) enzymes were determined using a microplate reader (Infinite M2000, Tecan, Männedorf, Switzerland), as described by Fu et al. [26]. BG is a C-acquiring enzyme, LAP and NAG are N-acquiring enzymes, and AP is a P-acquiring enzyme.

### 2.5. SOC Fractions

SOC fractions (POC and MAOC) were determined by the modified wet sieving approach, as described by Xu et al. [21]. Firstly, air-dried soil was mixed with sodium hexametaphosphate solution. Then, it was shaken at a speed of 180 r min^−1^ at 25 °C for 18 h to fully mix the reaction. And a 53 μm sieve was used to separate the slurries until the water flow was clear. The MAOC (<53 μm) and POC (>53 μm) were dried by oven-drying at 60 °C to a constant mass and weighed separately. The contents of SOC, MAOC, and POC were measured by using the potassium dichromate external heating method [33]. The recovery of the SOC was 90%.

### 2.6. Soil Respiration

The soil respiration was measured from the incubation as described by Qi et al. [34]. First, 20 g of fresh soil was placed into the bottom of a 300 mL culture bottle and then incubated with three blank controls (without soil addition) at 25 °C and 60% water holding capacity (WHC). In addition, 5 mL of NaOH (1 mol/L) solution was used to absorb CO_2_. Alkali traps were replaced at 1, 3, 5, and 7 days after incubation (Figure S1, Table S1), and then 0.20 mol/L HCI was used to titrate the NaOH solution. We found that after 7 days, CO_2_ emissions had stabilized, so we used the data from the previous 7 days. During the incubation, we keep the WHC using the weight method.

### 2.7. Statistical Analysis

Enzymatic vector analysis has been widely used to explore the response of microbial C and nutrient limitations to environmental changes [35,36].

x = BG: (BG + AP)(1)

y = BG: (BG + NAG + LAP)(2)

Vector length = SQRT(x^2^ + y^2^) (3)

Vector angle = degrees (Atan2 (x, y))(4)

The vector length indicates microbial C limitation. The vector angle indicates microbial P/N limitations (>45° is P-limited, and <45° is N-limited).

One-way analysis of variance (ANOVA) was used to analyze significant differences in the data among treatments and determined by the LSD test (*p* < 0.05) based on the IBM SPSS Statistics (version 26). We used the Pearson correlation analysis to determine correlations among SOC fractions (POC and MAOC) and soil properties, enzyme activity, and soil respiration. Random forest modeling was conducted to quantitatively assess the important contributions of soil properties, enzyme activity, and SOC fraction to soil respiration [37,38] by using the “randomForest”, “A3”, and “rfPermute” packages [39,40]. Partial least squares path models (PLS-PMs) were used to identify the direct and indirect effects of enzyme activity, SOC fractions, and soil properties on soil respiration in both rhizosphere and bulk soils (Table S2). The models were chosen with a goodness of fit (GoF) > 0.6 by using the “plspm” package [41,42]. All statistical analyses were carried out by R software (v.4.3.2).

## 3. Results

### 3.1. Intercropping Effects on Soil Respiration and SOC Fractions

Compared to monoculture, sugarcane–watermelon intercropping significantly increased SWC, TP, SAP, TN, and NO_3_^−^ content in both rhizosphere and bulk soils, and significantly decreased soil pH by ~0.60 units (Table S3).

**Figure 2:**
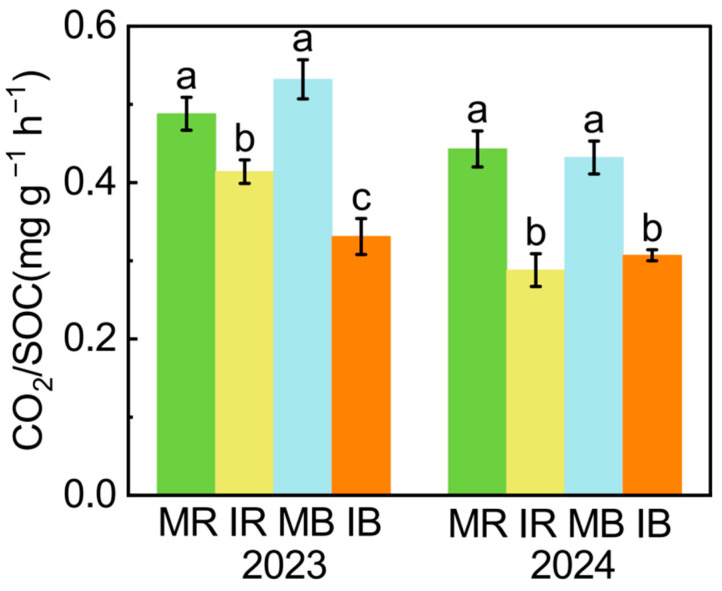
The intercropping effect on soil respiration in rhizosphere and bulk soil in 2023 and 2024. MR: monoculture rhizosphere; IR: intercropping rhizosphere; MB: monoculture bulk soil; IB: intercropping bulk soil. Different lowercase letters indicate significant differences among treatments, as determined by the LSD test (*p* < 0.05).

**Figure 3:**
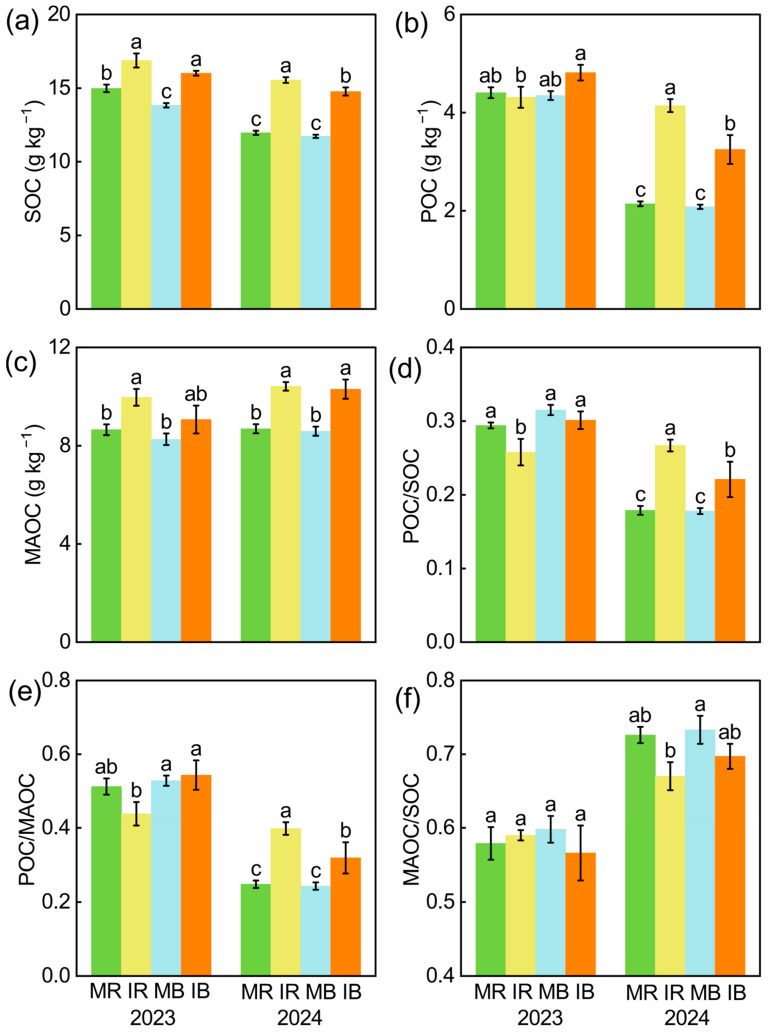
The intercropping effect on soil organic carbon SOC (**a**), particulate organic carbon (POC) (**b**), mineral-associated organic carbon (**c**), POC/SOC (**d**), MAOC/SOC (**e**), and MAOC/POC (**f**) in rhizosphere and bulk soil in 2023 and 2024. MR: monoculture rhizosphere; IR: intercropping rhizosphere; MB: monoculture bulk soil; IB: intercropping bulk soil. Different lowercase letters indicate significant differences among treatments, as determined by the LSD test (*p* < 0.05).

The sugarcane–watermelon intercropping decreased soil respiration (CO_2_/SOC), and this effect was higher in bulk soil than in rhizosphere soil (Figure 2). In addition, intercropping increased SOC and MAOC in all soils (Figure 3a,c). Compared to monoculture, the intercropping increased POC, POC/SOC, and POC/MAOC in 2024 and had few effects in 2023 (Figure 3b,d,e).

### 3.2. Intercropping Effect on Soil Enzyme Activities

**Figure 4:**
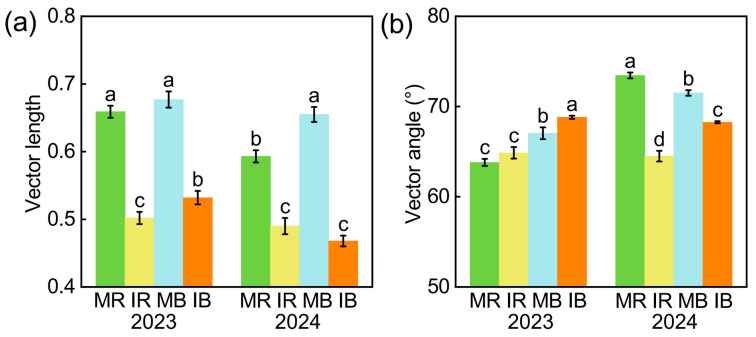
The intercropping effect on vector length (**a**) and vector angle (**b**) in rhizosphere and bulk soil in 2023 and 2024 years. MR: monoculture rhizosphere; IR: intercropping rhizosphere; MB: monoculture bulk soil; IB: intercropping bulk soil. The vector length indicates microbial C limitation. The vector angle indicates microbial P/N limitation. A vector angle > 45° suggests that microorganisms are P-limited. Different lowercase letters indicate significant differences among treatments, as determined by the LSD test (*p* < 0.05).

Intercropping increased NAG and AP contents in both rhizosphere and bulk soils (Figure S2a,d) and increased BG content solely in rhizosphere soil (Figure S2c). The vector length (microbial C limitation) declined with intercropping by 21~25% in rhizosphere and bulk soil (Figure 4a, *p* < 0.05). Microorganisms were P-limited with a vector angle above 45°, and the microbial P limitation was influenced by the cropping pattern, soil, and duration (Figure 4b).

### 3.3. Correlations Among Soil Respiration, Enzymes, and SOC Fractions

**Figure 5:**
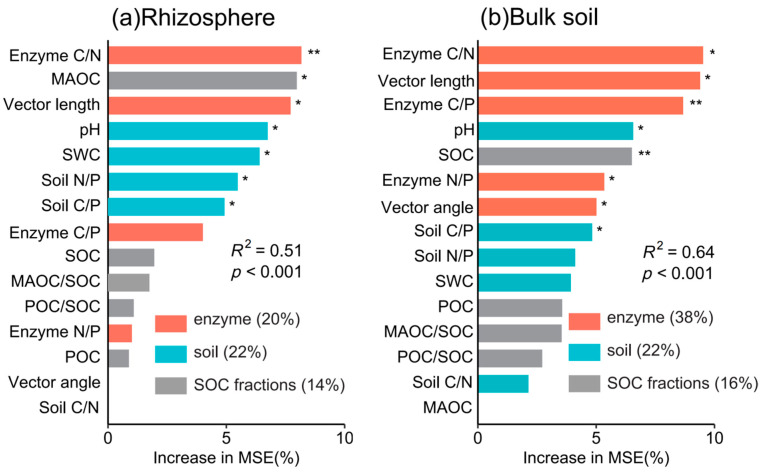
Relative importance of enzymes, soil properties, and SOC fractions for CO_2_/SOC in rhizosphere (**a**) and bulk soil (**b**). The relative importance of predictors is denoted by the percentage of increased mean square error (%MSE). SWC: soil water content; SOC: soil organic carbon; Soil C/N: the ratio of soil organic carbon to total nitrogen; Soil N/P: the ratio of soil total nitrogen to total phosphorus; Soil C/P: the ratio of soil organic carbon to total phosphorus; Enzyme C/N: BG/(NAG + LAP); Enzyme C/P: BG/AP; Enzyme N/P: (NAG + LAP)/AP; POC: particulate organic C; MAOC: mineral-associated organic C (*: *p* < 0.05; **: *p* < 0.01).

Pearson correlation showed that the contents of CO_2_ and CO_2_/SOC were significantly correlated with soil properties, enzyme activity, and SOC fractions (Figure S3). The random forest model ranked the relative importance of driving factors, with mean square error (%MSE) values quantifying their contributions to CO_2_/SOC variation (Figure 5). In rhizosphere soil, enzyme C/N, MAOC, vector length, soil pH, SWC, soil N/P, and C/P together explained 47% of CO_2_/SOC variation (Figure 5a). In bulk soil, enzyme C/N, vector length, enzyme C/P, soil pH, SOC, enzyme N/P, vector angle, and C/P accounted for 56% of the variability (Figure 5b). Among the different factors, enzyme activity was the most influential factor for CO_2_/SOC. SOC fraction factors are less significant than soil property factors.

**Figure 6:**
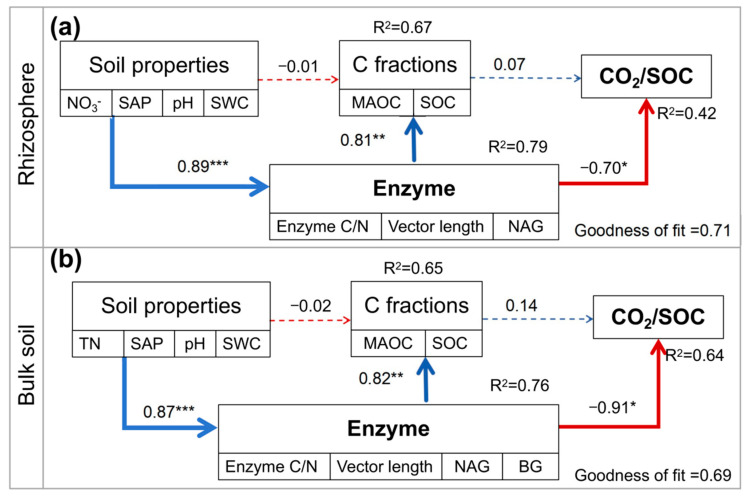
Partial least squares path modeling (PLS-PM) disentangling the major pathways of the influences of enzymes on soil respiration in the rhizosphere (**a**) and bulk soil (**b**). Blue and red arrows indicate positive and negative flows of causality, respectively. Numbers with arrows indicate significant standardized path coefficients. Dotted lines represent pathways with no significant effect. R^2^ indicates the variance of the dependent variable explained by the model (*: *p* < 0.05; **: *p* < 0.01; ***: *p* < 0.001). SAP: soil available phosphorus; TN: total nitrogen; NO_3_^−^: nitrate; SWC: soil water content; Enzyme C/N: BG/(NAG + LAP); NAG: β-1,4-Nacetylglucosaminidase; BG: β-1,4-glucosidase; SOC: soil organic carbon; MAOC: mineral-associated organic carbon.

The pathways were constructed to examine the effects of enzymes, soil properties, and SOC fractions on the variations of soil CO_2_ emission using the PLS-PM (Figure 6, Table S2). Intercropping significantly increased soil N and P content in both rhizosphere and bulk soil. Soil properties positively and directly affected soil C and N enzymes in both rhizosphere and bulk soil. Soil enzymes had positive direct effects on soil respiration in both rhizosphere and bulk soil. Moreover, soil enzymes had a positive direct effect on SOC fractions.

## 4. Discussion

### 4.1. Intercropping Reduced Soil Respiration

**Figure 7:**
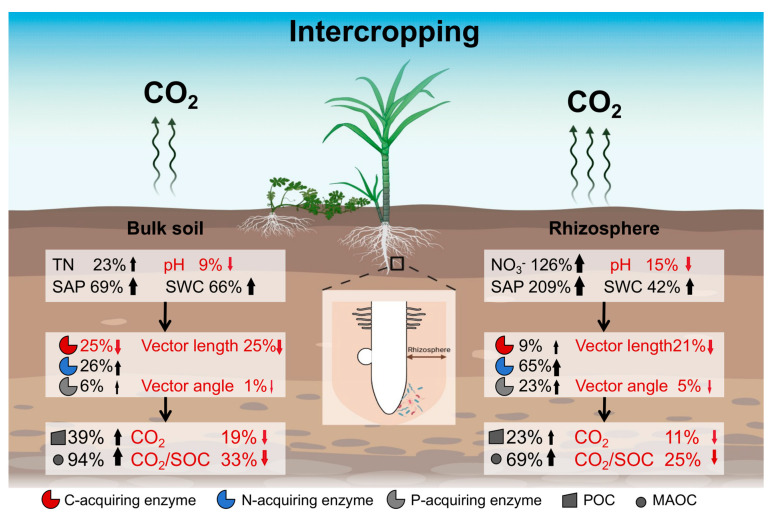
The regulatory mechanisms of rhizosphere and bulk soils on soil respiration in intercropping. Black and red arrows indicate the increase and decrease, respectively. SAP: soil available phosphorus; TN: total nitrogen; NO_3_^−^: nitrate; SWC: soil water content; POC: particulate organic carbon; MAOC: mineral-associated organic carbon.

We found that intercropping reduced soil respiration (CO_2_/SOC) by 25~33% (Figure 7, Table S1), which was similar to previous studies [43,44]. In the milk vetch-rapeseed intercropping system, soil CO_2_ emission was 35% lower than monoculture after the seedling stage because of a reduction in soil moisture [45]. Our results showed that intercropping increased soil moisture and nutrient availability (TP, SAP, TN, NO_3_^−^; Table S3), potentially contributing to reduced soil respiration. While previous studies suggest that the elevated nutrient availability can improve microbial carbon use efficiency (CUE), thereby suppressing respiration while promoting biomass synthesis [46,47,48], this mechanistic explanation remains speculative in our system. Direct evidence of whether intercropping-induced microbial CUE dynamics regulate soil respiration is currently lacking. Future investigations employing isotopic tracing (e.g., ^13^C-labeled substrates) to quantify CUE are needed to validate the intercropping effects on CUE. However, Wang et al. found that the intercropping effects on soil respiration were uncertain, with positive, negative, and even unchanged effects on CO_2_ emission in soybean–maize, wheat–soybean, and soybean–wheat intercropping, respectively [49]. This indicates that intercropping effects on soil respiration are dependent on crop types.

In addition, intercropping reduced the CO_2_/SOC by 25% in rhizosphere soil and 33% in bulk soil (Figure 7). This finding reveals that the intercropping effects on soil respiration are lower in the rhizosphere than in bulk soil [50]. This is because, in this study, the intercropping increased available nutrient content by 167% in the rhizosphere, which was higher than that in bulk soil (70%; Table S3). The increased nutrient availability enhances microbial metabolism [51], which offsets the negative intercropping effects on soil respiration to a larger extent in rhizosphere soil [52]. Therefore, intercropping is an effective approach to alleviate the greenhouse effect and has a great enhancement in soil C sequestration in agricultural soils. In subtropical red soil, the interplay of root exudates and mineral matrix changed the process of microbial decomposition [53]. The Fe minerals are positively charged and can sorb negatively charged carboxylic acid and some amino acids released [54]. Rhizosphere microbes, particularly Fe-reducing bacteria, employ siderophores and organic acids to solubilize Fe oxides, intentionally liberating adsorbed C as an energy source [55,56]. Concurrently, extracellular enzymes (e.g., peroxidases) adsorbed on mineral surfaces retain catalytic activity, enabling the gradual decomposition of residual organo-mineral complexes [57]. This interaction counteracts the expected decrease in CO_2_ emissions by the chemical protection of C in the rhizosphere.

### 4.2. Contributors to Soil Respiration with Intercropping

Microbial community diversity, microbial biomass, and soil nutrient availability influenced microbial metabolisms, which finally controlled the breakdown of soil organic matter and soil respiration [58,59,60]. In this study, microbial C limitation declined with intercropping (Figure 4a), and the declines in microbial C limitation reduced soil respiration and increased soil C sequestration (Figure 5 and Figure 6), which was similar to other studies [18,37,61]. Our recent study showed that intercropping sugarcane with peanuts can effectively alleviate microbial C limitation and nutrient limitation, which is consistent with the results of intercropping sugarcane with watermelon [26]. We speculate that there are two main reasons. On the one hand, intercropping increased root litter and exudates and soil available nutrients (Table S3). The increased available C content alleviated microbial C demands and less CO_2_ emission [62,63]. On the other hand, the increased soil nutrients promoted microbial C use efficiency, thereby reducing the secretion of enzymes by microorganisms [64]. Collectively, the decreased soil respiration was attributed to the reduction in microbial C limitation induced by high nutrient availability with intercropping. This implies the importance of microbial metabolism on soil C sequestration, and more long-term microbial observations should be considered in the future.

In addition, soil respiration had a negative correlation with soil SWC and a positive correlation with soil pH (Figure S3). The intercropping-induced high SWC and low pH reduced soil respiration through an influence on microbial C limitation (Table S3, Figure 6 and Figure S3). This is because the microbial community is sensitive to the changes in soil pH, with a shift from fungi-dominated to bacteria-dominated groups in low-pH soils [65]. The dominance of bacteria has fewer C demands and secretes fewer C-acquiring enzymes (low CO_2_ emission) as bacteria have lower biomass C/N than fungi [66]. High SWC reduced redox potentials with low O_2_ content, which inhibited microbial decomposition and CO_2_ emission. Therefore, the changes in soil pH and SWC induced by intercropping regulated microbial community and C limitation, which further controlled microbial decomposition and CO_2_ emission.

Additionally, the increased MAOC content reduced soil respiration (Figure S3), which was supported by previous studies [67,68]. This is because the turnover rate of MAOC was slow, which made it difficult for it to be decomposed and utilized [69]. The stabilization mechanisms of C exhibit striking divergence between subtropical and temperate soils, driven by distinct organo-mineral interactions and climatic constraints [70,71]. In subtropical red soils, MAOC formation is dominated by chemical adsorption processes, where Fe^3+^ and Al^3+^ oxides provide positively charged surfaces to immobilize labile organic compounds (e.g., root exudates) through ligand exchange or covalent bonding [72]. Consequently, despite high root exudation rates in subtropical ecosystems, the rapid adsorption of labile carbon onto Fe/Al oxides converts it into persistent MAOC, decoupling C inputs from CO_2_ emissions. In contrast, temperate soils primarily rely on physical protection mechanisms, where aggregates encapsulate POC, limiting microbial access [73]. However, this protection is transient and highly vulnerable to physical disturbances (e.g., tillage) or warming-induced microbial activation [74]. POC primarily consists of partially decomposed plant materials, which can be easily assimilated into microbes [75,76]. Intercropping improves POC content and microbial C use efficiency because of plant inputs [22,71]. The increased POC content usually leads to an increase in soil respiration, through high turnover [10,77]. However, intercropping improved the microbial C use efficiency, leading to the conversion of plant inputs to microbial biomass C [78,79], and reduced the correlation between POC and soil respiration. Therefore, intercropping-induced increased MAOC reduced CO_2_/SOC, especially in rhizosphere soil (Figure S3).

## 5. Conclusions

This study explored the intercropping effects on soil respiration and quantified the contributions of soil properties, enzyme activity, and SOC fractions to them based on long-term intercropping field experiments. Our findings showed that intercropping reduced soil respiration, with a greater reduction in bulk soils than rhizosphere soils, highlighting that the rhizosphere effect might offset the intercropping effect. This observation partly supported our original hypothesis. Additionally, the contents of SOC and MAOC were enhanced with intercrops. Sugarcane–watermelon intercropping regulated nutrients, pH, and SWC, in turn regulating soil C- and N-acquiring enzymes, and then influenced soil respiration and C sequestration. Our results imply that sugarcane–watermelon intercropping is a sustainable approach for decreasing soil respiration and contributes to soil C in agricultural soils.

## Data Availability

The original contributions presented in this study are included in the article/Supplementary Materials. Further inquiries can be directed to the corresponding author.

## Supplementary Materials

The following supporting information can be downloaded at: https://www.mdpi.com/article/10.3390/microorganisms13051049/s1.

## Abbreviations

The following abbreviations are used in this manuscript:

SOC	Soil organic carbon
PLS-PM	Partial least squares path model
MAOC	Mineral-associated organic carbon
POC	Particulate organic carbon
N	Nitrogen
P	Phosphorus
SOM	Soil organic matter
SWC	Soil water content
TN	Total nitrogen
NO_3_^−^	Nitrate
SAP	Soil available phosphorus
TP	Total phosphorus
BG	Β-1,4-glucosidase
AP	Acid phosphatase
NAG	Β-1,4-nacetylglucosaminidase
LAP	Leucine aminopeptidase
WHC	Water holding capacity
ANOVA	One-way analysis of variance
GOF	Goodness of fit
MR	Monoculture rhizosphere
IR	Intercropping rhizosphere
MB	Monoculture bulk soil
IB	Intercropping bulk soil
CUE	Microbial carbon use efficiency
