# Supplementary material for: Microbial Carbon Limitation Mediates Soil Organic Carbon Sequestration in Sugarcane–Watermelon Intercropping System
Source: Microorganisms. 2025 Apr 30;13(5):1049. doi: 10.3390/microorganisms13051049 (PMC12114252; doi:10.3390/microorganisms13051049)
Supplement: Supplementary file 1 [file microorganisms-13-01049-s001.zip › microorganisms-3577499-supplementary.pdf]

microorganisms

MDPI

Supplementary Materials:

Table S1. The ANOVA analysis of CO_2 and on CO_2/SOC among year, crop and soil.

Year	Soil	Cropping	CO_2 (mg kg h^{-1})	CO_2/SOC (mg g h^{-1})
2023	Rhizosphere	Monoculture	7.29±0.24a	0.49±0.02a
		Intercropping	6.96±0.16a	0.41±0.01b
	Bulk soil	Monoculture	7.37±0.40a	0.53±0.03a
		Intercropping	5.28±0.34b	0.33±0.02c
2024	Rhizosphere	Monoculture	5.23±0.23a	0.44±0.02a
		Intercropping	4.32±0.29b	0.29±0.02b
	Bulk soil	Monoculture	5.06±0.22ab	0.43±0.02a
		Intercropping	4.54±0.12ab	0.31±0.01b
	Year		***	***
	Cropping		***	***
	Soil		ns	ns
	Year*Cropping		ns	ns
	Year*Soil		ns	ns
	Cropping * Soil		ns	ns
	Year * Cropping* Soil		*	*

Values are means ± standard errors. Different lowercase letters indicate significant differences among treatments, as determined by the *LSD* test ($P < 0.05$), respectively. Significance level: ns: not significant; *: $P < 0.05$; **: $P < 0.01$; ***: $P<0.001$.

Table S2. Direct, indirect and total effects of rhizosphere and bulk. (Corresponding to Figure 5).

Soil	From	To	Direct effect	Indirect effect	Total effect
Rhizosphere	Soil properties	Enzyme	0.889	0.000	0.889
		C fractions	0.007	0.723	0.729
		CO_2/SOC	0.000	-0.578	-0.578
	Enzyme	C fractions	0.813	0.000	0.813
		CO_2/SOC	-0.704	0.053	-0.651
	C fractions	CO_2/SOC	0.007	0.000	0.070
Bulk soil	Soil properties	Enzyme	0.874	0.000	0.874
		C fractions	-0.020	0.718	0.698
		CO_2/SOC	0.000	-0.696	-0.696
	Enzyme	C fractions	0.821	0.000	0.822
		CO_2/SOC	-0.910	0.118	-0.793
	C fractions	CO_2/SOC	0.143	0.000	0.143

Table S3. The intercropping effect on soil properties in rhizosphere and bulk soil in 2023 and 2024 years.

Year	Treatment		SWC (%)	pH	TP (g kg^{-1})	SAP (mg kg^{-1})	TN (g kg^{-1})	NO_3^-–N (mg kg^{-1})
2023	Rhizosphere	Monoculture	16.08±0.07c	5.40±0.01a	0.60±0.01c	9.38±1.92b	1.08±0.03b	23.13±3.91b
		Intercropping	26.29±0.17a	4.79±0.03c	0.87±0.04b	34.43±6.85a	1.21±0.01a	58.62±5.57a
	Bulk soil	Monoculture	9.60±0.06d	5.16±0.02b	0.59±0.01c	12.71±3.53b	0.98±0.01c	23.81±6.00b
		Intercropping	20.76±0.33b	4.54±0.08d	0.98±0.02a	29.19±6.30a	1.19±0.01a	62.44±6.25a
2024	Rhizosphere	Monoculture	24.60±0.67b	5.48±0.04a	1.52±0.05c	27.24±3.54b	0.97±0.00c	24.96±0.91c
		Intercropping	29.50±0.44a	4.41±0.04d	2.30±0.02a	68.30±1.98a	1.33±0.00a	49.43±2.34a
	Bulk soil	Monoculture	22.38±0.46c	4.98±0.09b	1.45±0.02c	20.46±4.728b	0.99±0.01c	49.88±1.28a
		Intercropping	26.09±0.16b	4.72±0.13c	1.78±0.01b	22.04±2.33b	1.23±0.03b	38.87±1.05b

Values are means ± standard errors. SWC: soil water content; SOC: soil organic carbon; TP: total phosphorus; SAP: soil available phosphorus; TN: total nitrogen; NO_3^-–N: nitrate-nitrogen. Different lowercase letters indicate significant differences among treatments, as determined by the *LSD* test ($P < 0.05$), respectively.

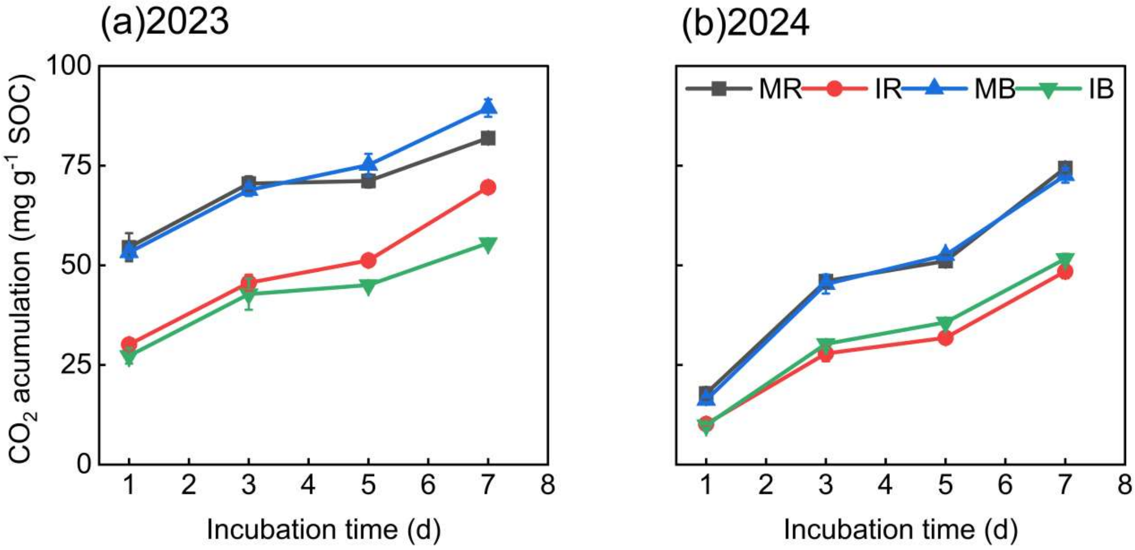

Figure S1. Total CO_2 accumulation over the 7-day of incubation period in 2023 and 2024 years. Values are the means ± standard errors (n = 6). MR: monoculture rhizosphere; IR: intercropping rhizosphere; MB: monoculture bulk soil; IB: intercropping bulk soil. The asterisk indicates significant effects of different treatments on the total CO_2 accumulation based on one-way ANOVA (*: $P < 0.05$; **: $P < 0.01$; ***: $P < 0.001$).

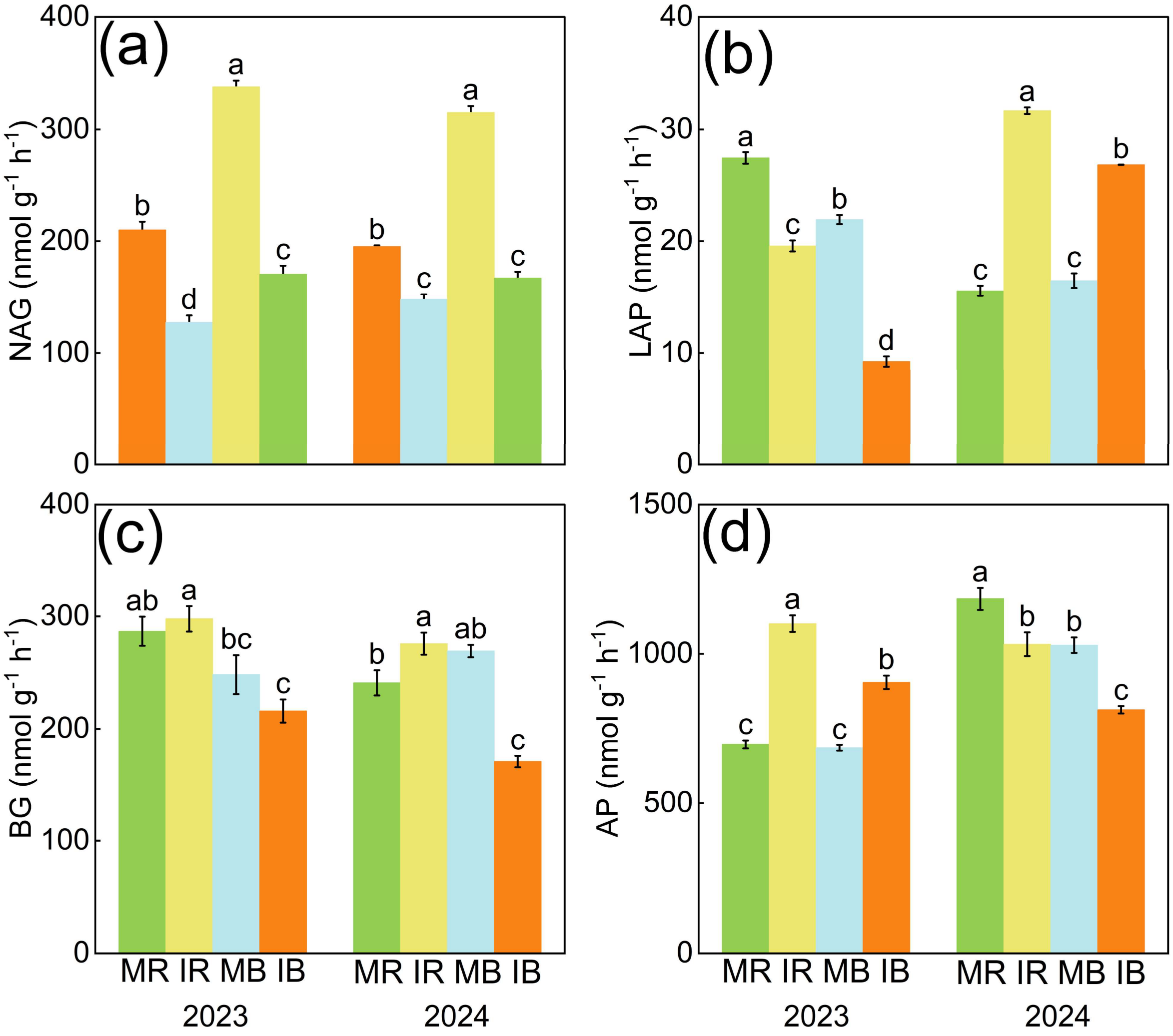

Figure S2. The intercropping effect on NAG enzyme activity (a), LAP enzyme activity (b), BG enzyme activity (c) and AP enzyme activity (d) in rhizosphere and bulk soil in 2023 and 2024 years. MR: monoculture rhizosphere; IR: intercropping rhizosphere; MB: monoculture bulk soil; IB: intercropping bulk soil; NAG: β-1,4-Nacetylglucosaminidase; BG: β-1,4-glucosidase; LAP: leucine aminopeptidase, AP: acid phosphatase. Different lowercase letters indicate significant differences among treatments, as determined by the *LSD* test ($P < 0.05$), respectively.

microorganisms

MDPI

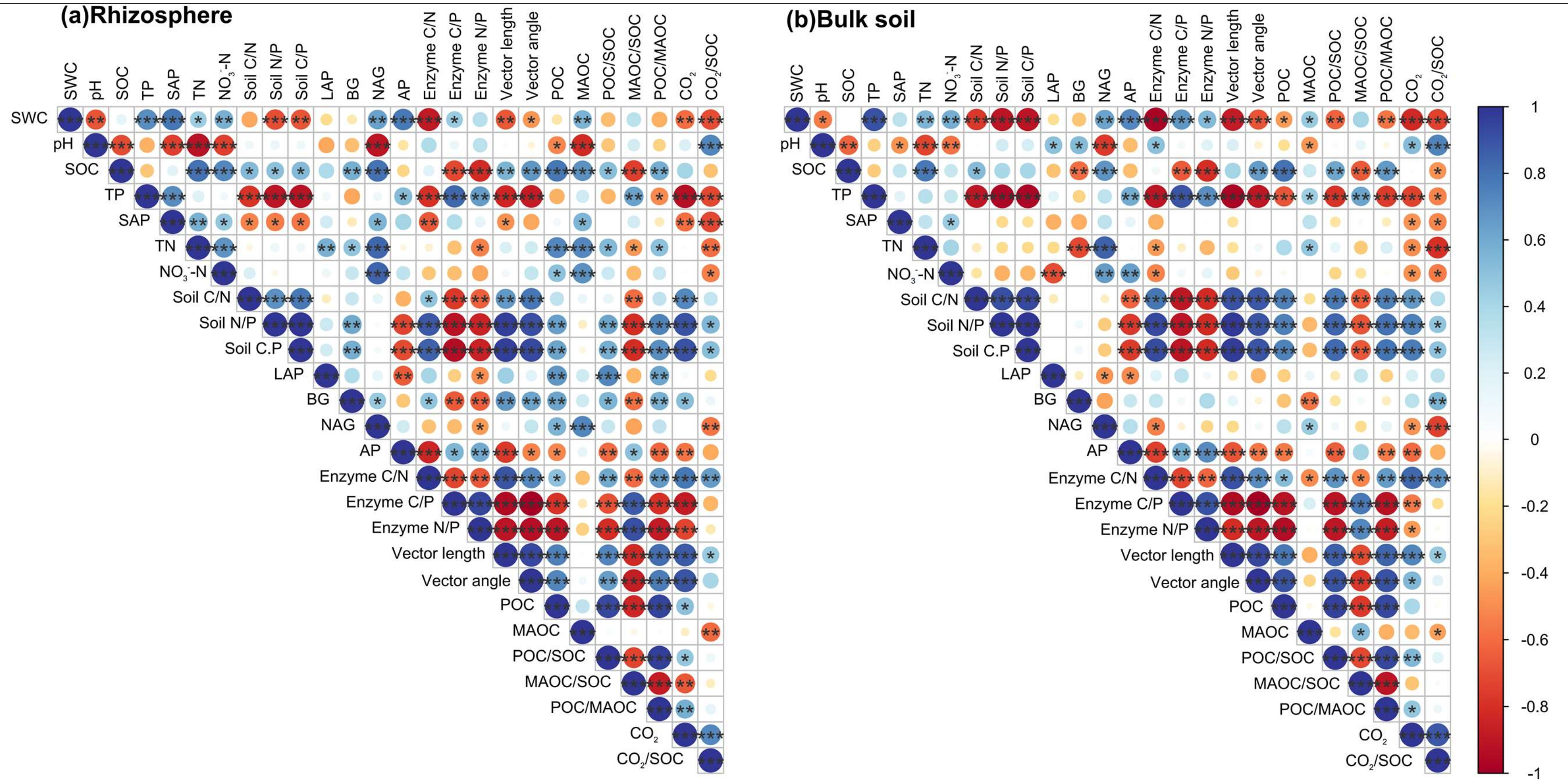

Figure S3. Pearson correlations among soil properties, SOC fractions, enzyme activity and soil respiration in rhizosphere (a) and bulk soil (b). Significance is indicated by asterisks (*: $P < 0.05$; **: $P < 0.01$; ***: $P < 0.001$). SWC: soil water content; SOC: soil organic carbon; TP: total phosphorus; SAP: soil available phosphorus; TN: total nitrogen; NO_3^-–N: nitrate-nitrogen; Soil C/N: soil organic carbon/ total nitrogen; Soil N/P: soil total nitrogen/ total phosphorus, Soil C/P: soil organic carbon/total phosphorus; NAG: β-1,4-Nacetylglucosaminidase; BG: β-1,4-glucosidase; LAP: leucine aminopeptidase; AP: acid phosphatase; Enzyme C/N: BG/(NAG+LAP); Enzyme C/P: BG/AP; Enzyme N/P: (NAG+LAP)/AP; POC: particulate organic C; MAOC: mineral associated organic C.
